# Aqueous two-phase system to isolate extracellular vesicles from urine for prostate cancer diagnosis

**DOI:** 10.1371/journal.pone.0194818

**Published:** 2018-03-27

**Authors:** Hyunwoo Shin, Yong Hyun Park, Yong-Goo Kim, Ji Youl Lee, Jaesung Park

**Affiliations:** 1 Department of Mechanical Engineering, POSTECH, Pohang, Republic of Korea; 2 Department of Urology, Seoul St. Mary’s Hospital, The Catholic University of Korea, Seoul, Republic of Korea; 3 Department of Laboratory Medicine, Mary’s Hospital, The Catholic University of Korea, Seoul, Republic of Korea; University of South Alabama Mitchell Cancer Institute, UNITED STATES

## Abstract

Analyzing extracellular vesicles (EVs) is an attractive approach to diagnosis of prostate diagnosis. However, existing methods of EVs isolation have low efficiency, purity, and long process time, and therefore have low diagnostic ability. To solve these the problems, a two-phase system is adapted to isolate EVs from a patient’s urine. Urine from 20 prostate cancer (PCA) patients and 10 benign prostate hyperplasia patients was used to quantify the EVs-isolation ability of an aqueous two-phase system (ATPS) and to compare the diagnostic ability of ATPS with that of the conventional diagnosis method. An optimized ATPS isolates EVs with ~100% efficiency within ~30 min, with 14 times as high as achieved by ultracentrifugation. Afterward, PCR and ELISA are used to detect EVs derived from PCA cells in urine. The results demonstrate that diagnostic ability based on ATPS is better than other conventional diagnostic methods. ATPS can obtain a high quality and quantity of EVs from patients’ urine. EVs contain cancer-related protein and genes, so these abundant sources enable diagnosis with high specificity and sensitivity. Therefore, ATPS is a useful tool to increase the specificity and sensitivity of diagnosis.

## Introduction

Serum prostate-specific antigen (PSA) is a marker that is widely used to detect incipient cancer, and to provide a post-treatment prognosis. However, PSA can also increase during benign hyperplasic conditions, so PSA does not exhibit sufficient diagnostic specificity [1]. To overcome this limitation of PSA as a disease marker, approaches that analyse urinary sediments or extracellular vesicles (EVs) have been introduced [2–4]. Urinary sediments may contain a few red blood cells, white blood cells, epithelial cells, and microorganisms. Prostate cancer (PCA) cells secrete proteins into urine in a way similar to circulating tumour cells (CTCs) passing barriers between tumour and body vessels, and the sediments can contain PCA cells originated from tumour tissues, as well [5, 6]. Although the sediments where CTCs are contained may be used for cancer diagnosis based on RNA or protein analysis, the sediments are expected to contain only a few cancer cells, as CTCs are extremely rare [7]. Consequently, urinary sediments is deficient in cancer-associated RNA or protein sources, and require efficient CTC isolation methods for use in PCA diagnosis.

EVs secreted by cells offer major advantages, such as abundance, stability and diversity, for the purpose of cancer diagnosis [8]. Large quantities of EVs have been detected in several body fluids, including blood, ascites and cerebrospinal fluid, whereas CTCs are rare and are therefore not identified [9–11]. However, EVs vary greatly in size (50 nm–500 nm) and biological variety, so their use for clinical purposes presents a unique set of challenges. Currently, EVs are isolated by a variety of methods, such as ultracentrifugation (U/C), size exclusion chromatography and immunoaffinity. These methods commonly involve a lengthy and complicated process with low yield, so they have compromised the utility of EVs for clinical diagnosis [12–23].

Use of aqueous two-phase systems (ATPSs) can overcome these difficulties of isolating EVs. ATPSs exploit the incompatibility of two aqueous phases of polymeric molecules. ATPSs partition different kinds of particles into the two phases in a short time. ATPSs have been used to separate cells with different membrane-surface properties, to separate proteins, and to improve the sensitivity of polymerase chain reaction (PCR) detection by extracting PCR inhibitors [24–28].

In this study, a polyethylene glycol (PEG)/ dextran (DEX) ATPS was used to isolate EVs from human urine. After EVs were isolated from patients’ urine, prostate hyperplasia (BPH) patients and PCA patients were detected by quantifying mRNA and protein expression levels. Compared to diagnosis methods based on urine sediments and serum PSA, the proposed method successfully differentiates PCA from BPH with higher specificity and selectivity.

## Materials and methods

### Preparation of human samples

Twenty patients who had PCA and 10 patients who had benign prostate hyperplasia (BPH) were recruited, and their Gleason score, tumor stage, and serum-PSA (S1 Table) were measured using biopsies. From each patient, a 10-ml sample of the urination in the morning was collected in a sterile container, then preserved at -80 °C until they were used in the experiments. The urine was centrifuged at 2,000 × g-force for 20 min at -4 °C. The supernatant was isolated, then stored in a new tube at -80 °C. Sediment was suspended in 100 μl of phosphate-buffered saline (PBS) and stored at -80 °C. All procedures used in the experiment were approved by the Ethics Committee of South Korea (IRB number: KC14SISI0213), and all experiments were performed in accordance with the approved guidelines and regulations. Each participant gave written consent.

### EV isolation with ATPS and U/C

Optimized PEG/DEX ATPS were adapted for EV isolation from urine. ATPSs were prepared by dissolving the polymers directly in 5 ml of urine at 4 °C for 1 h. The solution became opaque when the polymer was completely dissolved. The solutions were vortexed, then separated into phases by centrifugation at 1,000 × g for 10 min at 4 °C. After separation, the top phase and bottom phase could be distinguished by the presence of an interface. The top phase was composed of a PEG-rich solution, and the bottom phase was composed of a DEX-rich solution. During centrifugation the EVs migrated to the DEX-rich phase. The PEG-rich phase was carefully extracted using a pipette, and transferred into a new tube. The solution near the phase interface was removed separately using a pipette. The remaining DEX-rich phase was transferred into a different tube for further analysis.

To increase the purity of the isolated EVs, a serial protein depletion process was used [29]. Briefly, a polymer solution was prepared by dissolving PEG and DEX in PBS in the same composition as in the system used to isolate EVs. The solution was centrifuged at 1,000 × g for 10 min, then the PEG phase was collected. This PEG phase in the purification solution had the same polymer composition as the PEG phase of the urine samples. After the first phase separation in urine, 4 ml of the (top) PEG phase was carefully removed without touching the interface. The same volume of purification solution was then added to the remaining (bottom) DEX-phase and interface, and the sample was mixed and centrifuged at 1,000 × g for 10 min. These steps were repeated, and the recovery efficiency of the EVs and the purity of the isolated EVs in each step were measured. After two repetitions of the steps twice, the recovery efficiency and purity ceased to increase, so we used just two steps in downward assays [30].

EVs were also isolated from the urine by using “U/C-once” and “U/C-twice” methods to compare their isolation efficiency. In the U/C-once method, 60 ml of the urine was diluted with 5 ml of PBS containing EDTA (final concentration 5 mM), then ultra-centrifuged at 100,000 × g for 2 h. The supernatant was then discarded, and the EV pellet was resuspended in 100 μl of PBS for further analysis. In the U/C-twice method, the resuspended 100 μl solution was diluted with 4 ml of PBS, ultra-centrifuged again at 100,000 × g for 2 h, then processed in the same way as the U/C-once method.

Urine contains both EVs and proteins, so measurement of total protein content cannot be used to quantify EVs. Instead, EVs were quantified by measuring the RNA content of the samples. Pre- and post-isolation samples from the ATPS and U/C were lysed using 0.8 ml TRI Reagent (Sigma Aldrich) for 5 min at room temperature. Then 0.2 ml of chloroform was added to the lysed samples, and they were centrifuged at 16,100 × g for 10 min to separate the phases. Aqueous phase containing RNA was carefully extracted, and an equal volume of isopropyl alcohol (IPA) was added to it to precipitate the RNA. This aqueous phase/IPA mixture was then centrifuged at 16,100 × g for 10 min to pelletize the RNA. The supernatant was discarded, the RNA pellet was washed with 75% ethanol, and then centrifuged at 13,500 × g for 10 min. Finally, the washed RNA was dissolved in 20 μl of nuclease-free water, and the amount of RNA was measured using a spectrophotometer (Genway, Genova) to quantify the amount of EV in the sample. The amounts of protein in the pre- and post-isolation samples were quantified using Bradford protein assays.

### Protein- and RNA-based assays using EVs isolated by ATPS and U/C

Western blot, enzyme-linked immunosorbent assay (ELISA), qualitative RNA profile analysis, and polymerase chain reaction (PCR) were performed to compare the accuracy of protein- and RNA-based assays using EVs isolated by ATPS and U/C. In western blot, total EVs isolated by U/C and ATPS from 5 ml urine were used, and EV-specific markers (tetraspanin proteins CD9, CD81, and CD63) were identified. Qualitative RNA profile analysis was applied to the total RNA extracted from EVs isolated by U/C and ATPS from 5 ml urine. In PCR, the total RNA was amplified, and house-keeping genes (actin and GAPDH) were identified.

### Prostate cancer diagnosis using serum-PSA, sediments, and EVs by ATPS

**Fig 1:**
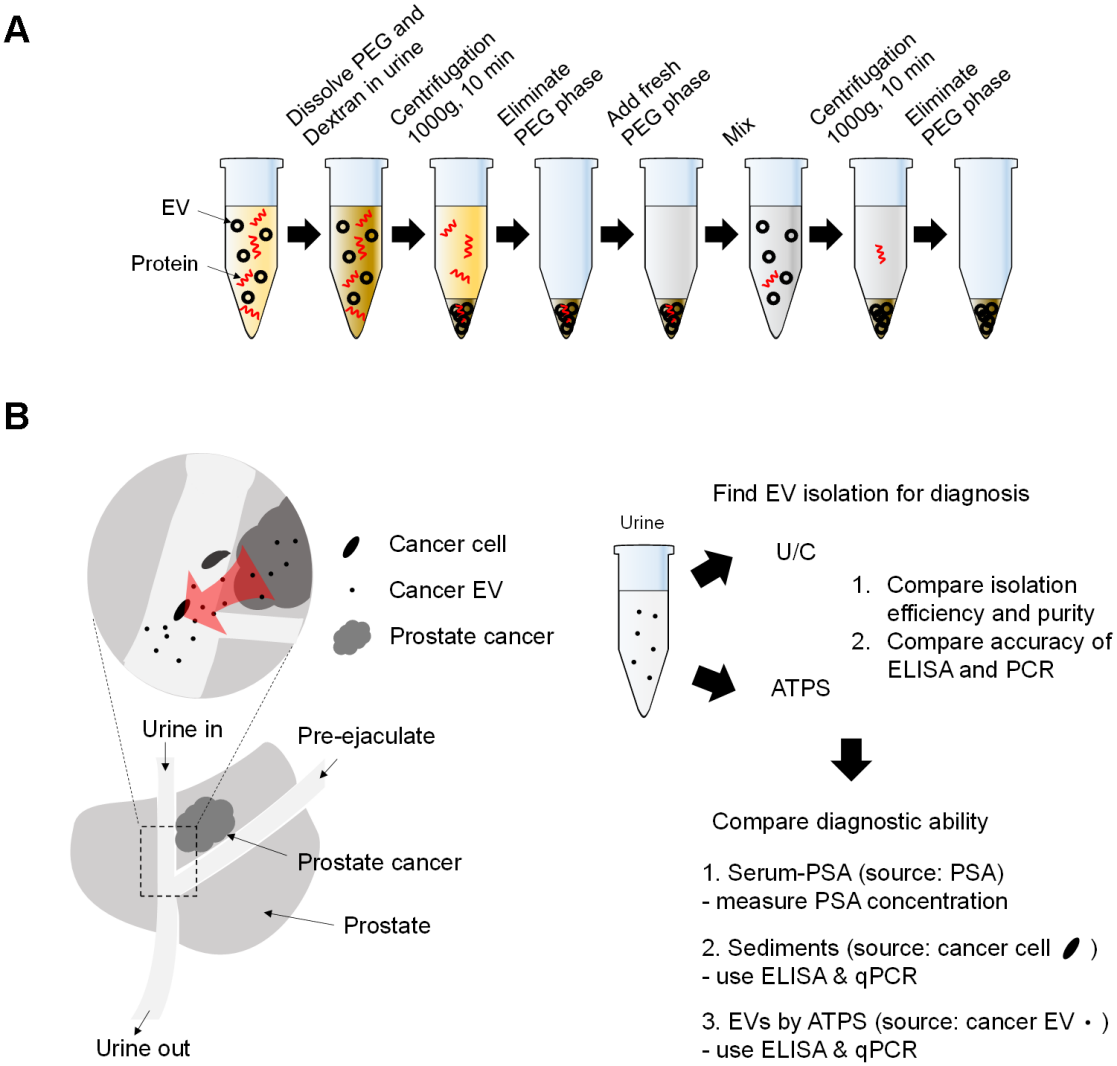
Scheme of experiments. (**A**) Use of ATPS to isolate EVs in urine. Red particles: proteins; black particles: EVs. After the polymers are dissolved, the solution forms an ATPS, and particles segregate depending on their surface property to one of the phases. Subsequent purification steps yield highly pure EVs. (**B**) Cancer cells and EVs in prostate release to urine. EVs are isolated by ATPS from 5 ml urine. The diagnostic ability of serum-PSA, sediments, and EVs was compared using samples from 20 patients who have prostate cancer (PCA) and 10 patients who have benign prostate hyperplasia (BPH).

Optimized ATPS was used for PCA diagnosis. (Fig 1A) [29]. Diagnosis using EVs isolated by the ATPS, and two conventional diagnosis methods (serum-PSA and sediments) were performed (Fig 1B) [1, 31]. After using ATPS to isolate EVs from the patients’ urine, their expression of genes associated with PCA was quantified and compared with conventional methods (sediments and serum PSA) [1, 31]. Prostate-specific membrane antigen (PSMA) is a PCA-associated protein marker, and PCA antigen 3 (PCA3) is a PCA-specific gene marker [32, 33]. Accordingly, EVs derived from PCA cells are hypothesized to contain PSMA and PCA3 [3].

Patients’ states were identified by biopsy, then their serum-PSA concentrations were measured by immunometric assay using kits (Tandem-R) obtained from Hybritech. We prepared 5-ml urine samples from the patients, centrifuged the samples at 2000 × g for 20 min, then precipitated the sediments. The supernatant was transferred to another tube for use in isolating EVs, and the pellet was dissolved in 100 μl of PBS. Diagnosis using sediments was performed by ELISA and quantitative polymerase chain reaction (qPCR). In ELISA, PSMA in the sediments was measured and normalized by the creatinine concentration ([creatinine]) in urine to represent its dilution factor [34, 35]. Urinary [creatinine] was measured by Jaffe's method using a commercial kit obtained from Cobas. In qPCR, the expression level of PCA3 in sediments, was measured and normalized by actin. PCA3 is highly expressed in PCA cells.

EVs of urine were collected only by ATPS for diagnosis because the ATPS isolates more and higher-quality EVs than U/C does. In the same way, the concentration of PCA-specific protein marker prostate-specific membrane antigen (PSMA) in the EVs was measured and normalized by [creatinine] of urine. Additionally, PSMA in the EVs was normalized by CD9, which represents the amount of EVs. In qPCR, the expression level of PCA antigen 3 (PCA3) in EVs, which is highly expressed in PCA, was measured and normalized by actin.

The diagnostic ability of each method was compared by drawing a receiver operating characteristic curve (ROC) using IBM SPSS Statistics 21 (IBM).

### Western blot

Western blot was processed in the conventional method. Briefly, 2 μl of DEX-rich phase and centrifuged EVs sample was mixed with 38 μl of distilled water and 10 μl of 5x SDS-PAGE loading buffer (250 mM Tris–HCl, 10% SDS, 0.5% bromophenol blue, 50% glycerol). The mixtures were boiled at 100 °C for 10 min, separated using SDS polyacrylamide gel electrophoresis (12% resolving gel, 120 V, 90 min), and then transferred to a PVDF membrane at 390 mA for 2 h, at 4 °C. The transferred PVDF membrane was treated with blocking solution (3% non-fat milk, PBS), then treated with 0.1 mg/ml CD9, CD81, and CD63 primary antibody (Santa Cruz, Armenian Hamster Anti-Mouse, diluted with blocking solution). Then 0.1 mg/ml HRP conjugated secondary antibody (Santa Cruz, anti-hamster IgG-HRP) in blocking solution was applied for 1 h, and the presence of target protein was detected by adding chemi-luminescent substrate (Femto, Amersham Pharmacia Biotech).

### Enzyme-linked immunosorbent assay (ELISA)

The CD9 Exo ELISA Kit (EXOEL-CD9A-1, System Biosciences, Mountain View, CA, USA) was used for measurement of EV level according to the manufacturer's instructions. PSMA level in EVs from human plasma was determined using the human glutamate carboxypeptidase 2 (FOLH1) ELISA kit (MBS901525, MY BioSource, Inc., San Diego, CA, USA) according to the manufacturer's instructions.

### Qualitative RNA profile analysis

The qualitative profiles of previously-prepared RNA samples were analyzed using an Agilent RNA 6000 Nano Kit (Agilent Technology) according to the manufacturer’s instructions.

### Polymerase chain reaction

Reverse-transcript PCR was performed using a GoScript reverse transcription kit (Promega) and amplified using a GoTaq polymerase chain reaction kit (Promega) following the manufacturer’s protocol. The sequences of primers used in PCR were: Actin forward 5’-AGA GCT ACG AGC TGC CTG AC-3’ reverse 5’-AGC ACT GTG TTG GCG TAC AG -3’. Actin was quantified using qPCR. cDNA samples were used as the template in a qPCR reaction (20 μl final volume) containing 0.2 μM inner primer and 1X Power SYBR Green Master Mix (Applied Biosystems). The qPCR program entailed thermo-cycles of 94 °C for 5 min; 40 cycles of 94, 55 and 72 °C for 30 s each; then 72 °C for 10 min.

For nested quantitative PCR, initial rounds of amplification were performed with the GoScript reverse transcription kit (Promega) and amplified using the GoTaq polymerase chain reaction kit (Promega) following the manufacturer’s protocol. The sequences of primers used in PCR were: PCA3_outer forward 5’-AGT CCG CTG TGA GTC T-3’ reverse 5’-CCA TTT CAG CAG ATG TGT GG-3’, and PCA3_inner forward 5’-ATC GAC GGC ACT TTC TGA GT-3’ reverse 5’TGT GTG GCC TCA GAT GGT AA-3’. PCA3 was quantified using nested qPCR, and actin was quantified using qPCR. The PCR program comprised thermo-cycles of 94 °C for 5 min; 15 cycles of 94, 52 and 72 °C for 30 s each; and then 72 °C for 10 min. Amplified DNA samples were used as the template in a qPCR reaction (20 μl final volume) containing 0.2 μM inner primer and 1X Power SYBR Green Master Mix (Applied Biosystems). The qPCR program entailed thermo-cycles of 94 °C for 5 min; 40 cycles of 94, 52 and 72 °C for 30 s each; then 72 °C for 10 min.

### Nanoparticle tracking analysis

Nanoparticle tracking analysis (NTA) was used to count the EVs. Samples treated using ATPS or U/C were placed in the chamber of a Nanosight LM10 (Malvern Instruments, Ltd.) and analyzed using NTA software (Malvern, Nanosight software version 2.3).

### Transmission electron microscopy (TEM)

To visualize and examine the morphology of isolated EVs, TEM was performed; 5 μl of each isolated sample was deposited on a formavar carbon film (FCF300-cu, Electron Microscopy Science), then mixed with 7 μl of 2% uranyl acetate for 10 s to stain it. Samples were air-dried for 30 min, and then imaged at 60-kV acceleration voltage on a transmission electron microscope (JEM-1011, Japan).

### Statistical analysis

All data were presented as means and standard deviations. Statistical analyses were performed using IBM SPSS Statistics 21 (IBM). Data were first analyzed using ANOVA, and then differences between pairs of means were tested using Tukey’s test. Two-way ANOVA was performed to identify interactions between EVs and proteins.

## Results

### EV isolation

**Fig 2:**
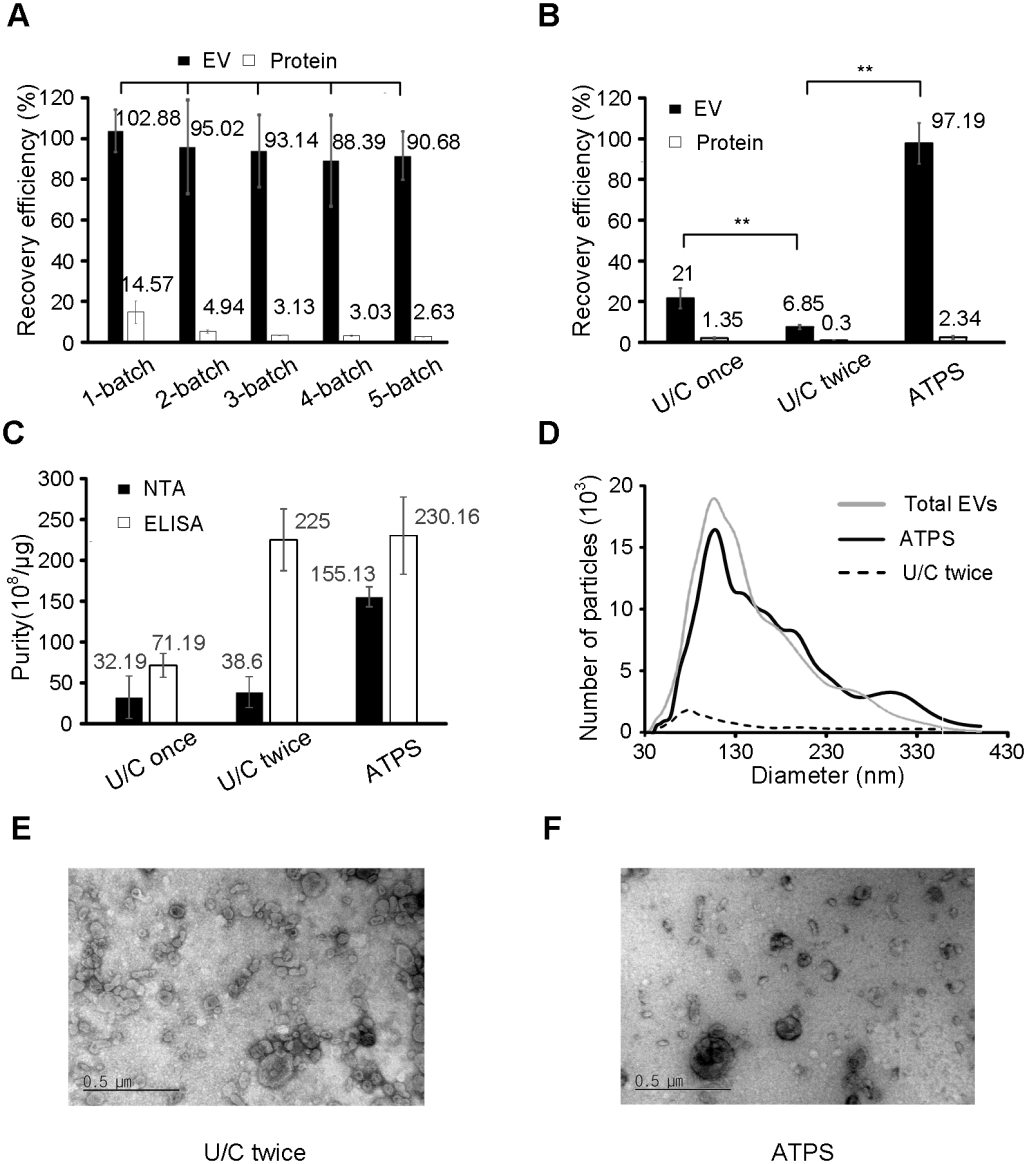
Characterization and conventional identification of ATPS compared to conventional methods. (**A**) Additional purification steps of serial protein-depletion. After the procedure, the recovery efficiency of EVs was almost unchanged, whereas the recovery efficiency of proteins decreased. (**B**) Recovery efficiency of EVs and protein in U/C-once, U/C-twice, and ATPS. Recovery efficiency of EVs by ATPS was four times larger than by U/C-once, and 14 times larger than by U/C-twice. (**C**) Purity of EVs in U/C-once, U/C-twice, and ATPS. Purity is the number of isolated EVs divided by amount of isolated protein. Amount of EVs was measured by NTA and ELISA. Protein was measured by Bradford assay. (**D**) Size distribution of EVs isolated from urine by U/C-twice and ATPS was measured using NTA. Size distributions of total EVs in 5 ml urine: grey line; black line, estimated using ATPS; dotted line, estimated using U/C-twice. Grey line and black line are similar; i.e., ATPS isolated ~100% of EVs. U/C twice isolated only a small fraction of EVs. (**E**, **F**) TEM did not show any morphological differences between EVs isolated using U/C-twice and ATPS. The results were analyzed by ANOVA with a post-significance Tukey’s test. pairwise significances: *p < 0.05, **p <0.001.

During ATPS, most of the EVs and ~15% of the proteins from the urine migrated into the DEX phase (Fig 2A). To minimize protein impurity for efficient downstream analysis, we performed serial protein depletion processes that were first introduced in PBS-based solution [29]. PEG phase was repeatedly replaced by fresh PEG extracted proteins from the DEX phase. After the PEG phase had been replaced five times, the amount of proteins in the bottom DEX phase decreased to one-fifth of that after single ATPS. However, the quantity of EVs was nearly unchanged, and the recovery efficiencies of EVs in each protein-depletion process (PDP) were not statistically different. After the third PDP, additional PDPs did not significantly decrease protein-recovery efficiency, so we isolated EVs from urine by ATPS after the third PDP. The decrease in protein-extraction efficiency after the third PDP may be a consequence of strong interaction between the DEX phase and hydrophilic surfaces.

To evaluate ATPS EV-isolation efficiency, it was compared with U/C-once and U/C-twice. The ATPS isolation method optimized by adjusting polymer concentration recovered approximately 100% of EVs from the urine, whereas the U/C-once recovered 21% and UC-twice recovered 6.85% (Fig 2B). Protein recovery partially represents the amounts of impurities. ATPS recovered 2.34% of protein, whereas U/C-once recovered 1.35% and U/C-twice recovered 0.3%, but the relative purity ((EV-recovery efficiency)/(protein-recovery efficiency)) of ATPS was 41.5, which greatly exceeded those of U/C-once (15.5), and U/C-twice (22.8). This result implies that ATPS isolates a higher quality and quantity of EVs than do existing methods.

ATPS achieved greater EV purity (*P* = (total number of EVs)/(total amount of protein)) than U/C once and U/C twice (Fig 2C). NTA results suggest that ATPS achieved *P* = 155.13×10^8^/μg, U/C-once achieved *P* = 32.19×10^8^/μg, and U/C-twice achieved *P* = 38.6×10^8^/μg. ELISA results suggest that ATPS achieved *P* = 230×10^8^/μg U/C-once achieved *P* = 71.19×10^8^/μg, and U/C- twice achieved *P* = 225×10^8^/μg.

NTA results also indicate that ATPS achieved higher the recovery efficiency of EVs than the other methods (Fig 2D). The size distribution profiles of EVs in initial urine and EVs isolated by ATPS were similar, and corresponded to the recovery efficiency (~97%) calculated by RNA amounts (Fig 2B). In contrast, the number of EV isolated by U/C-twice was smaller than the total number of EVs in initial samples, and the recovery efficiency of particles calculated by NTA was <10%, which is similar to the RNA-based result (Fig 2B). EVs isolated using ATPS did not differ morphologically from EVs obtained using U/C (Fig 2E and 2F).

### Compatibility with diagnostic purpose

**Fig 3:**
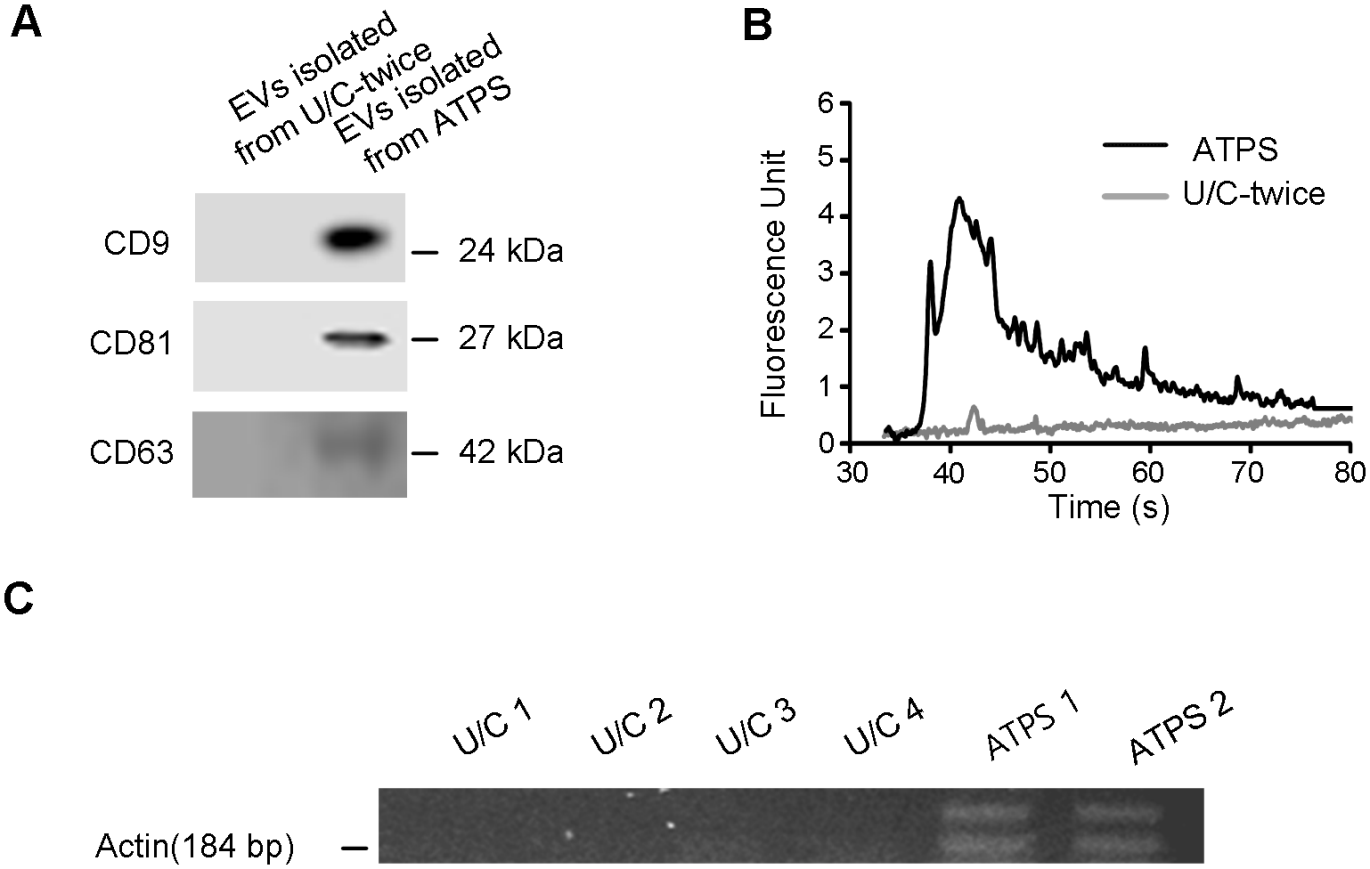
ELISA and PCR of EVs isolated by ATPS and U/C-twice. (**A**) Existence of EV surface marker was analyzed by CD9, CD81, and CD63 western blot. Total protein isolated from 5 ml urine using ATPS and U/C-twice was used for ATPS and U/C-twice samples. Final volume of isolated EVs samples by the methods was 250 μl, and 40 μl of the samples were used in western blots; 1.5 μg (ATPS) and 0.2 μg (U/C-twice) of protein was used in each well. (**B**) RNA profile comparison between ATPS and U/C-twice. The total RNA isolated from 5 ml urine using ATPS and U/C was compared. RNA profiles of ATPS and U/C differed significantly. (**C**) PCR of actin was performed using RNA extracted from EVs isolated by ATPS and U/C-twice from 5 ml urine. Isolated total RNA was used for PCR; ~800 ng (ATPS), and ~ 50 ng (U/C 1, 2, 3, and 4) of RNA was used.

To compare EVs isolated by ATPS and U/C-twice, protein markers of EVs and RNA were analyzed (Fig 3). Western blotting was performed using total EVs isolated by these methods from the same initial 5 ml of urine (Fig 3A and S1 Fig). EV surface markers CD9, CD81 and CD63 were detected easily in the EVs isolated by ATPS, but were not detected in EVs isolated by U/C-twice because the band signal was too weak. The difference was induced by recovery efficiency (ATPS ~97%, U/C- twice ~7%). However, if the quantity of EVs obtained using UC-twice was increased by 14 times, strong bands of CD9, CD81, and CD63 were observed, similar to those of ATPS. This result confirmed the reason that that the bands of U/C-twice were not detected is that the method has low isolation efficiency.

To examine the possibility of diagnostic application using RNAs in EVs, RNA profile and PCR using EVs isolated by the methods were performed. For EVs isolated by ATPS and using U/C-twice from 5 ml urine, RNAs were isolated and compared using a bio-analyzer (Fig 3B). The bio-analyzer detected a large amount of RNA in ATPS, but could not detect RNA contents of U/C-twice because of its poor isolation efficiency (6.8%) and the small volume of urine (5 ml). These results indicate that ATPS provides better RNAs than U/C-twice, and that RNAs isolated from EVs using ATPS can be used as a biomarker with only 5 ml of urine.

PCR was performed using total RNA extracted from EVs isolated by ATPS and U/C-twice from 5 ml urine (Fig 3C and S2 Fig). EVs isolated by ATPS yielded a strong actin band, but EVs isolated by U/C-twice showed no such band. These results may be due to the different recovery efficiency of the methods (ATPS: 97.19%; U/C-twice: 6.85%). ATPS provided sufficiently high recovery efficiency for the downstream analyses, but the low recovery efficiency and purity of EVs in U/C-twice reduced the effectiveness of protein- and RNA-based assays, which were conventionally used for diagnosis. Some assays may not be possible with EVs isolated by U/C-twice, due to its low recovery efficiency.

### Diagnosis of prostate cancer

**Fig 4:**
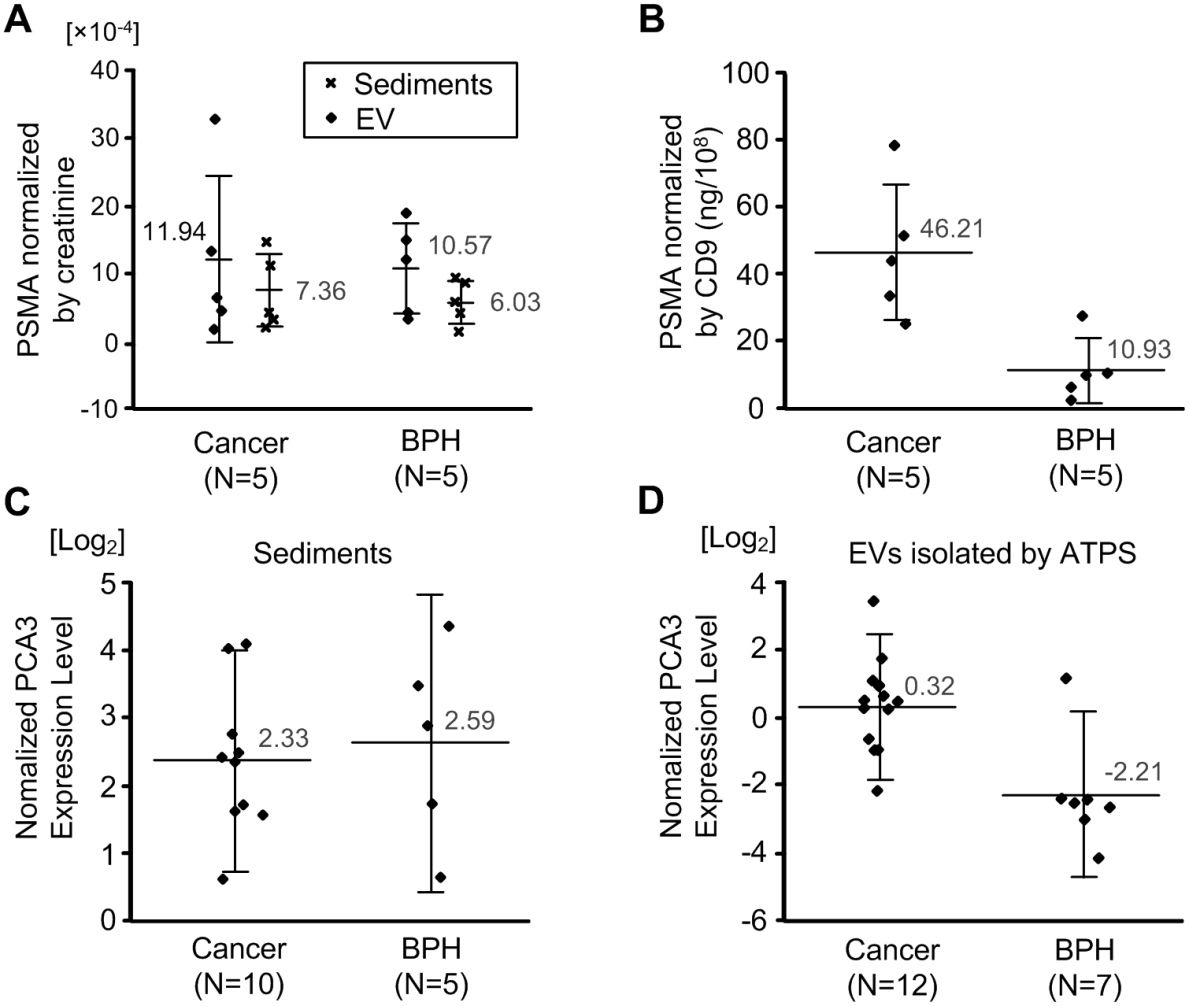
Prostate cancer-associated gene expression using ATPS and sediments. (**A**) Amounts of prostate-specific membrane antigen (PSMA) (normalized by creatinine concentration in urine) obtained using sedimentation and EVs isolated by ATPS in prostate cancer (PCA) and prostate hyperplasia (BPH) patients. (**B**) Amounts of prostate-specific membrane antigen (PSMA) (normalized by CD9) obtained using EVs isolated by ATPS in PCA and BPH groups. (**C**) RNA expression level of prostate cancer antigen 3 (PCA3) (normalized by actin) obtained using sedimentation in PCA and BPH groups; y-axis scale is log2. Large value means that PCA3 was highly expressed. (**D**) RNA expression level of PCA3 (normalized by actin) in EVs obtained using ATPS, in PCA and BPH groups. qPCR was performed to measure gene expression levels.

After isolating sediments and EVs from 5 ml of patients’ urine, the amount of PSMA was measured and normalized by [creatinine] of the urine to reduce the variable concentration of the urine samples (Fig 4A). The average value of PSMA/[creatinine] was slightly higher in the cancer group than in the BPH group in both sediments and EVs (sediments: cancer, 7.36, BPH, 6.03; EVs: cancer, 11.94, BPH, 10.57the), but the differences were not statistically significant because of a wide standard deviation (p > 0.1). However, differences between the groups were significant when PSMA was normalized by CD9, which is an EV-specific marker (cancer, 46.21, BPH, 10.93, p < 0.05) (Fig 4B). This result indicated that PSMA expression was higher in EVs from cancer patients than from BPH patients.

For RNA-based diagnosis, expression levels of PCA3 and actin were measured by applying qPCR to sediments and EVs, and the gene expression level of PCA3 was normalized by actin, which is a house-keeping gene (Fig 4C and 4D). Although the same gene markers (PCA3 and actin) were used in both diagnoses using sediments, the normalized expression levels of PCA3 were clearly different. In EVs, PCA3/actin was 0.32 in cancer patients, but -2.21 in BPH patients. In sediments, PCA3/actin was 2.33 in cancer patients, but 2.59 in BPH patients. In EVs, the average value of expression levels was higher in the cancer group than in the BPH group. However, differences between the groups were significant only in EVs (p<0.05). In both assays, diagnosis was better using EVs than using sediments.

**Fig 5:**
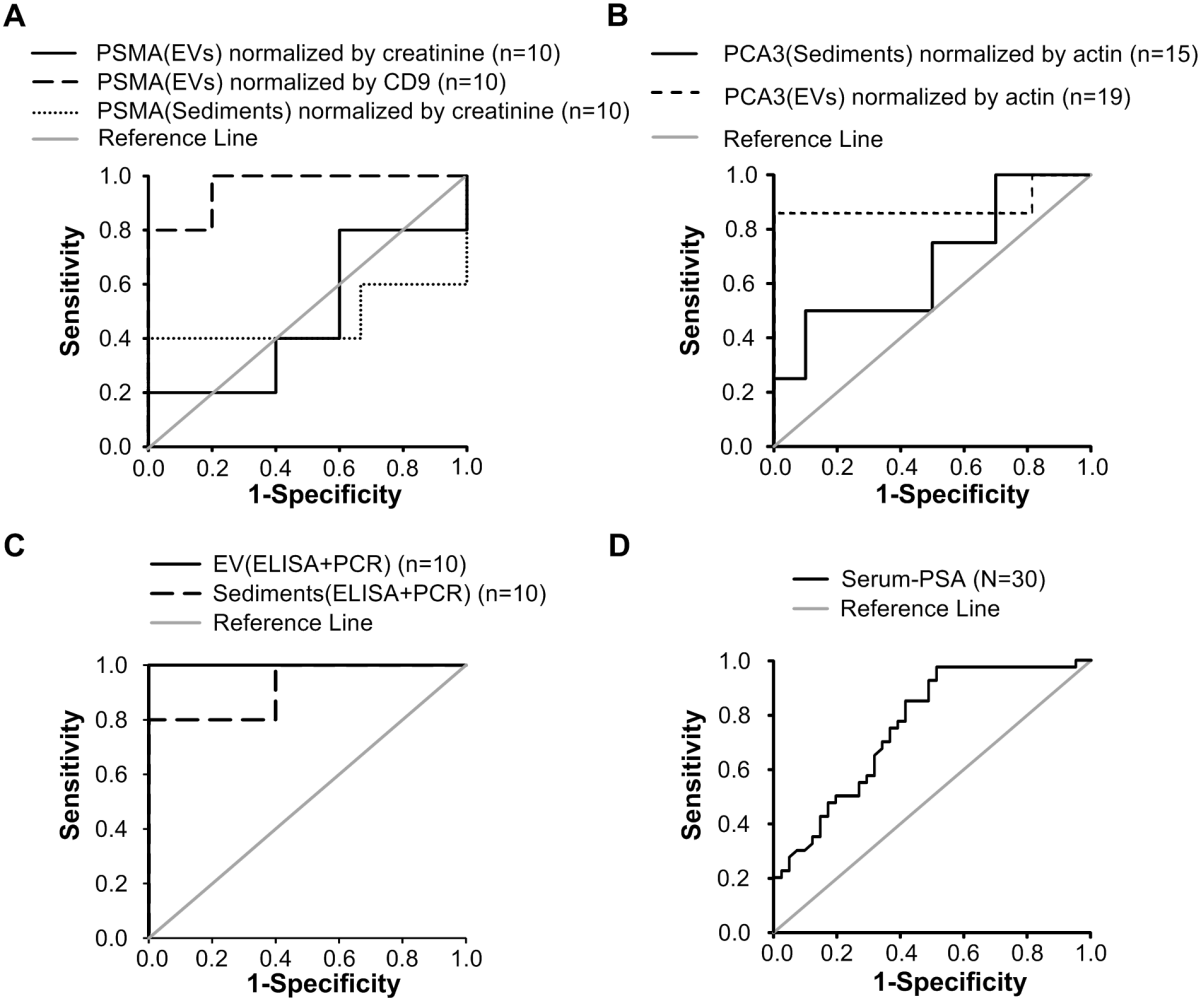
Diagnostic ability of conventional methods and ATPS. (**A**) Receiver operating characteristic (ROC) curve based on ELISA for diagnosis using ATPS and sediments. PSMA/creatinine or PSMA/CD9 was measured for diagnosis. (**B**) ROC curve based on qPCR for diagnosis using ATPS and sediments. PCA3/actin was measured for diagnosis. (**C**) ROC curve for diagnosis by combining ELISA and qPCR using ATPS and sediments. (**D**) ROC curve for serum-PSA.

Sensitivity and specificity of differential diagnosis of PCA and BPH using protein (PSMA) in EVs were assessed using receiver operating characteristic (ROC) curves (Fig 5A). The area of PSMA of EVs normalized by CD9 (PSMA/CD9 of EVs) was larger than the other diagnoses, which means that the diagnosis using PSMA/CD9 of EVs provides better diagnostic reliability than the other methods. Assessments of sensitivity and specificity of diagnosis using RNA (PCA3) of EVs were also performed in the PCA and the BPH group using a receiver operating characteristic (ROC) curve; diagnosis using EVs was also better than using sediments (Fig 5B). When diagnosis using protein and RNA was combined, the diagnostic ability was improved in both EVs and sediments (Fig 5C). The ROC curve of serum PSA diagnosis indicates that serum PSA diagnosis provides lower diagnostic ability than diagnosis using EVs and sediments combining RNA and protein (Fig 5D).

Using the ROC curve, the areas under the curve (AUC), sensitivity (true positive rate), and specificity (true negative rate) of the diagnosis methods were measured (Table 1). Diagnosis using EVs obtained using ATPS correctly distinguished all 10 PCA patients from the BPH patients when RNA and protein were combined. In both sediments and EVs, combining protein and RNA for diagnosis achieved a notable improvement in AUC, sensitivity and specificity of diagnosis. When data from sediments were used, protein achieved AUC = 0.467, RNA achieved AUC = 0.657 and protein + RNA achieved AUC = 0.92. In EVs, protein achieved AUC = 0.96, RNA achieved AUC = 0.883, and protein + RNA achieved AUC = 1.

**Table 1:** AUC, sensitivity, and specificity of methods to distinguish PCA from BPH.

Method	Marker	AUC	Sensitivity	Specificity
Serum PSA	Protein (PSA) (n = 30)	0.759	0.7	0.659
Sediments	Protein (PSMA/creatinine) (n = 10)	0.467	0.4	0.667
	RNA (PCA3/actin) (n = 15)	0.657	0.75	0.675
	**Protein+RNA (n = 10)**	**0.92**	**0.8**	**1**
EVs	Protein (PSMA/creatinine) (n = 10)	0.48	0.8	0.4
	Protein (PSMA/CD9) (n = 10)	0.96	1	0.8
	RNA (PCA3/actin) (n = 19)	0.883	0.857	0.909
	**Protein+RNA (n = 10)**	**1**	**1**	**1**

Sensitivity and specificity were also improved by combining protein and RNA in sediments and EVs. The diagnosis accuracy was higher in the method that uses protein + RNA of EVs than in the method that uses sediments. This result implies that protein and RNA of EVs originated from PCA were more abundant than sediments. As a result, use of ATPS instead of serum PSA for diagnosis increased the true positive rate by 30%, and the true negative rate by 34.1%. ATPS increased the true positive rate by 20% compared to the sedimentation method.

## Discussion

We isolated EVs using DEX/PEG ATPS. Isolation of EVs in ATPS can be affected by: (1) van der Waals interaction force; (2) hydrogen bonding; (3) electrostatic interaction; (4) polymer and ion binding or repulsion; and (5) hydrophobic and hydrophilic interaction. Although those factors cannot be decoupled successfully, and analysis of factors that drive EVs separation is not straightforward, the main contributing factors are expected to be hydrophobic- and hydrophilic interactions because the other factors can be reasonably ruled out: (1) van der Waals interaction forces mainly affect separation at high polymer concentration, but the polymer concentration of the ATPS is < 10%; (2) hydrogen bonding forces between EV and PEG or EV and DEX are both weak; (3 and 4) the polymers that comprise ATPS do not have any charge and cannot be ionized.

Hydrophobic and hydrophilic interactions are favored because the DEX phase is more hydrophilic than the PEG phase, so the EVs partition into the DEX phase. In addition, most EVs membranes are hydrophilic because they are derived from the cell membrane. We demonstrated the advantages of a high-yield EV-isolation method that uses a concentration-adjusted PEG/DEX ATPS. When diagnosing PCA, the largest advantages of ATPS are efficiency, purity, and simplicity. Biased affinity of EVs to DEX-rich phase results in recovery efficiency approximately 14 times higher than what was achieved by U/C-twice. The different affinities of EVs and proteins to each phase separates these particles, and makes high purity possible.

The important advantage of ATPS is that it does not need any special equipment. Simply adding polymers to sample solution causes formation of an ATPS, and EVs move spontaneously to the DEX-rich phase. We used centrifugation to accelerate phase separation, this step is optional because gravity induces phase separation. In short, to obtain a large amount of highly purified EVs, the only necessary process is addition of polymers.

These advantages facilitate research based on EVs; for example, diagnostic applications. In clinical diagnosis, accuracy and processing time determine the practicality of diagnosis methods. Sensitivity and specificity of disease markers are related to accuracy, and simplicity is associated with processing time. The proposed method increases the sensitivity and specificity of known markers (PCA3 and PSMA) by using a large amount of high-purity EVs. Furthermore, use of ATPS simplifies the the assay protocol for PCA.

Because ATPS isolates a large quantity of high-quality EVs from patients’ urine, a large amount of genes and protein derived from PCA can be obtained. We performed diagnosis using both gene and protein markers (PCA3 and PSMA). Diagnosis had poor sensitivity and specificity when a single marker was used, but the diagnostic ability was increased by combining gene and protein markers; this combination reduced false diagnosis by >30% compared to conventional diagnostic methods. Therefore, ATPS offers a powerful tool for specific and sensitive diagnosis. We used only two kinds of markers for PCA diagnosis, so we expect that diagnostic ability would be improved by combining more markers.

However, diagnosis based on various markers is labor-intensive and time-consuming. The isolation of EVs itself only takes about 5% of total process time; the other 95% is consumed by PCR and ELISA. Thus, for use of EVs as a diagnosis tool to be clinically useful, simplified marker-detecting processes must be developed.

## Supporting information

S1 Fig Full images of blots in Fig 3A. Cropped image within the manuscript is indicated in black box. Existence of EV surface marker was analyzed by CD9, CD81, and CD63 western blot. The total protein isolated from 5 ml urine using ATPS and U/C-twice was used for ATPS and U/C-twice samples. Final volume of isolated EVs samples by the methods was 250 μl, and 40 μl of the samples were used in western blots; 0.2 μg (U/C-twice) and 1.5 μg (ATPS) of protein was used in each well. EV surface marker CD9, CD81, and CD63 were detected easily in the EVs isolated by ATPS, whereas those markers were not detected in the EVs isolated by U/C-twice because the band signal was too weak.
(TIF)

S2 Fig Full images of gels in Fig 3C. Cropped image within the manuscript is indicated in white box. PCR of actin was performed using RNA extracted from EVs isolated by ATPS and U/C-twice from 5 ml urine. Isolated total RNA was used for PCR; approximately 50 ng (U/C 1, 2, 3, and 4), approximately 800 ng (ATPS) of RNA was used.
(TIF)

S1 Table Characteristics of the cancer patients. (TIF)
