# Supplementary figures and images for: Aqueous two-phase system to isolate extracellular vesicles from urine for prostate cancer diagnosis
Source: PLoS One. 2018 Mar 27;13(3):e0194818. doi: 10.1371/journal.pone.0194818 (PMC5870972; doi:10.1371/journal.pone.0194818)

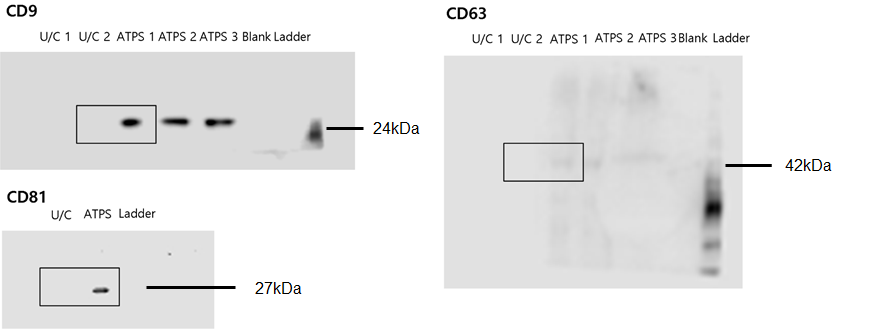

Supplement: S1 Fig — Cropped image within the manuscript is indicated in black box. Existence of EV surface marker was analyzed by CD9, CD81, and CD63 western blot. The total protein isolated from 5 ml urine using ATPS and U/C-twice was used for ATPS and U/C-twice samples. Final volume of isolated EVs samples by the methods was 250 μl, and 40 μl of the samples were used in western blots; 0.2 μg (U/C-twice) and 1.5 μg (ATPS) of protein was used in each well. EV surface marker CD9, CD81, and CD63 were detected easily in the EVs isolated by ATPS, whereas those markers were not detected in the EVs isolated by U/C-twice because the band signal was too weak. (TIF) [file pone.0194818.s001.tif]

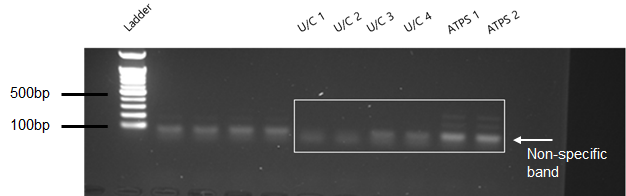

Supplement: S2 Fig — Cropped image within the manuscript is indicated in white box. PCR of actin was performed using RNA extracted from EVs isolated by ATPS and U/C-twice from 5 ml urine. Isolated total RNA was used for PCR; approximately 50 ng (U/C 1, 2, 3, and 4), approximately 800 ng (ATPS) of RNA was used. (TIF) [file pone.0194818.s002.tif]

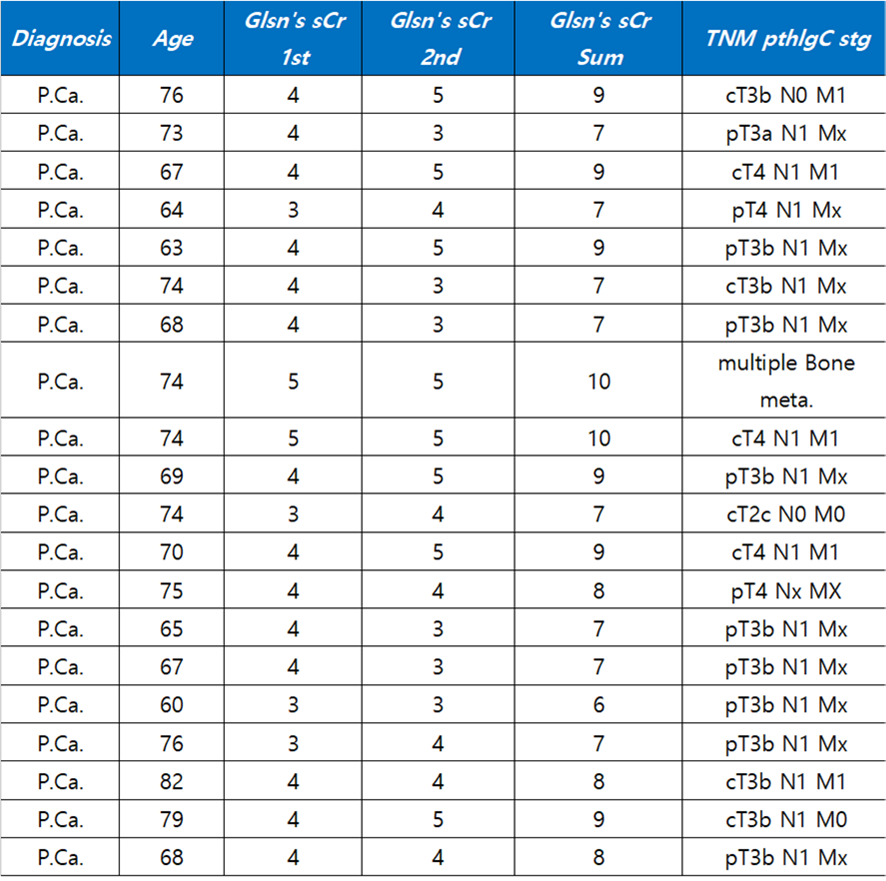

Supplement: S1 Table — (TIF) [file pone.0194818.s003.tif]
